# Activation of Molecular Signatures for Antimicrobial and Innate Defense Responses in Skin with Transglutaminase 1 Deficiency

**DOI:** 10.1371/journal.pone.0159673

**Published:** 2016-07-21

**Authors:** Takashi Haneda, Yasutomo Imai, Ryosuke Uchiyama, Orie Jitsukawa, Kiyofumi Yamanishi

**Affiliations:** 1 Department of Dermatology, Hyogo College of Medicine, Nishinomiya, Hyogo, Japan; 2 Department of Microbiology, Hyogo College of Medicine, Nishinomiya, Hyogo, Japan; CNRS-University of Toulouse, FRANCE

## Abstract

Mutations of the *transglutaminase 1* gene (*TGM1*) are a major cause of autosomal recessive congenital ichthyoses (ARCIs) that are associated with defects in skin barrier structure and function. However, the molecular processes induced by the transglutaminase 1 deficiency are not fully understood. The aim of the present study was to uncover those processes by analysis of cutaneous molecular signatures. Gene expression profiles of wild-type and *Tgm1*^–/–^epidermis were assessed using microarrays. Gene ontology analysis of the data showed that genes for innate defense responses were up-regulated in *Tgm1*^–/–^epidermis. Based on that result, the induction of *Il1b* and antimicrobial peptide genes, *S100a8*, *S100a9*, *Defb14*, *Camp*, *Slpi*, *Lcn2*, *Ccl20* and *Wfdc12*, was confirmed by quantitative real-time PCR. A protein array revealed that levels of IL-1β, G-CSF, GM-CSF, CXCL1, CXCL2, CXCL9 and CCL2 were increased in *Tgm1*^–/–^skin. Epidermal growth factor receptor (EGFR) ligand genes, *Hbegf*, *Areg* and *Ereg*, were activated in *Tgm1*^–/–^epidermis. Furthermore, the antimicrobial activity of an epidermal extract from *Tgm1*^–/–^mice was significantly increased against both *Escherichia coli* and *Staphylococcus aureus*. In the epidermis of ichthyosiform skins from patients with *TGM1* mutations, S100A8/9 was strongly positive. The expression of those antimicrobial and defense response genes was also increased in the lesional skin of an ARCI patient with *TGM1* mutations. These results suggest that the up-regulation of molecular signatures for antimicrobial and innate defense responses is characteristic of skin with a transglutaminase 1 deficiency, and this autonomous process might be induced to reinforce the defective barrier function of the skin.

## Introduction

Autosomal recessive congenital ichthyoses (ARCIs) are rare hereditary skin disorders, in which abnormal skin with generalized scales and desquamations develop [1]. The two major types of ARCI are lamellar ichthyosis (LI) and congenital ichthyosiform erythroderma (CIE). LI is characterized by brownish thick plate-like scales, while CIE shows erythroderma with whitish scales of various sizes. Bathing suit ichthyosis (BSI) is a rare minor subtype of ARCI, in which the trunk of the body rather than the extremities is mainly affected. Self-improving collodion ichthyosis or self-healing collodion baby and acral self-healing collodion baby are also other minor subtypes of ARCI, in which thick scales occur during a limited time and areas of the skin in infancy. Mutations in the *transglutaminase 1* gene (*TGM1*), which encodes transglutaminase 1 (TGM1), are most frequently identified in those major and minor subtypes of ARCI [1].

TGM1 is a member of the transglutaminase family (EC 2.3.2.13) that catalyzes the cross-linking between polypeptides via ε-(γ-glutamyl) lysine bonds. In normal epidermis, TGM1 is induced in the upper spinous and granular layers and is anchored to the plasma membrane to assemble the cornified envelope (CE) at the cell periphery of corneocytes [2, 3]. The CE is composed of thickly polymerized proteins and plays an important role as a strong barrier against physical, chemical and microbial invasions [4]. TGM1 also has an activity to cross-link ω-hydroxyceramides to involucrin, a component of the CE [5]. Indeed, *Tgm1* knockout (*Tgm1*^–/–^) mice [3, 6] and *Tgm1*^*R142C/R142C*^ mice with homozygous mutations of R142C in the enzyme [7] show a defective CE and have disorganized stratum corneum intercellular lipid molecules with severe skin permeability barrier defects. The pathology of *Tgm1*^–/–^mice and an ARCI patient with a TGM1 null mutation shows epidermal acanthosis with a severely thickened stratum corneum [3, 8]. Those particular phenotypes of TGM1 deficiency possibly develop in response to defects in cutaneous barrier structure and function [6]. However, the molecular mechanisms leading to the variety of phenotypes in ARCI with TGM1 mutations, often accompanied by cutaneous inflammation presenting as erythema or erythroderma, are largely unknown.

Skin barrier defects easily permit the invasion of microbial pathogens into the skin, but nevertheless it is not common that patients with LI or CIE have severe cutaneous infections. Possibly, some molecular processes are induced to control cutaneous infections in those ichthyoses. More than 20 types of antimicrobial peptides (AMPs) take part in the cutaneous innate immune system through their antimicrobial and chemoattractant activities or as proteinase inhibitors. The abnormal expression of those proteins influences the pathogenesis of various skin disorders, such as psoriasis, atopic dermatitis and rosacea [9]. However, the activation of AMPs and innate immune responses in ARCIs has not been studied before. In the present study, we report that the molecular signatures of antimicrobial and innate defense responses are activated in the skin of *Tgm1*^–/–^mice and in an ARCI patient with *TGM1* mutations. The activation of those genes may be an important autonomous process to reinforce the defective skin barrier function in TGM1 deficiencies.

## Materials and Methods

### Human specimens

The use of human specimens for this research was reviewed and approved by the Ethics Committee of the Hyogo College of Medicine (Permit Number: 212). Written informed consent was obtained from each patient or donor and all research was conducted according to the principles expressed in the Declaration of Helsinki.

### Animals

The study design followed the International Guiding Principles for Biomedical Research Involving Animals published by the Council for the International Organization of Medical Science. Studies using mice were reviewed and approved by the Animal Use and Care Committee of the Hyogo College of Medicine (Permit Number: B09-251; B09-305; B10-085; B11-023; 13–001; 15–067). Mice were maintained under specific pathogen-free conditions. *Tgm1*^+/–^mice [3] with a C57BL/6 background were intercrossed to generate *Tgm1*^–/–^mice. Primers MhomoU (5'-GGGAATGCTGGTTGTGACTGGTGTGGAT-3') and L972-2 (5'-GCGTAGGTTTAGGTTGTGTCCGTTGTTCTTAG-3') were used for genotyping of *Tgm1*^–/–^mice. For sampling specimens, pregnant mice and pups were euthanized by cervical dislocation under anesthesia with pentobarbital and hypothermia, respectively, to minimize suffering.

### Isolation of epidermis

Dorsal skin of 19.5 day post-coitum (dpc) mice was excised and washed in phosphate buffered saline (PBS). Subcutaneous tissue was removed from each specimen and the skin was incubated in PBS containing 10 mM EDTA at 37°C for 1 h. The epidermis was gently separated from the dermis with fine forceps and was used for the preparation of RNA or protein extracts.

### Isolation of RNA

Tissue specimens were immersed in RNA*later*^®^ RNA Stabilization Solution (Thermo Fisher Scientific Inc., Waltham, MA) at 4°C overnight and were stored at -20°C. Total RNA from each specimen was prepared using a RNeasy Fibrous Tissue Kit (Qiagen, Inc., Hilden, Germany) according to the manufacturer’s instructions.

### Microarray and data mining

Microarray analysis of epidermal RNAs using an Agilent SurePrint G3 Mouse GE 8x60Kv.1 (Agilent Technologies, Santa Clara, CA) was outsourced to Takara Bio Inc. (Mie, Japan). Data of the microarrays were deposited at the NCBI’s Gene Expression Omnibus under accession number GSE81109. The raw data were imported into GeneSpring software (Agilent Technologies) and were processed by log2 transformation and normalization of 75% shift. Data from low quality entities flagged with “not detected” and/or “compromised” were removed and data between the 20 to 100 percentile were retained. Nine entities of data (ID_REF: A_55_P2011877; A_51_P402994; A_30_P01023652; A_30_P01022001; A_30_P01032945; A_30_P01030803; A_30_P01020783; A_52_P113537; A_52_P300376) simply related to sex were also removed. A total of 3,704 entities were changed more than 2-fold on average. Of those, 630 entities were altered more than 5-fold and Gene ontology (GO) in those conditions was assessed using GeneSpring. The probability of each GO term was estimated by a standard hypergeometric distribution and a corrected-P value was calculated using the Benjamini Yuketieli procedure. Networks of the listed entities were analyzed using natural language processing algorithm (NLP) in GeneSpring, in which single and direct interactions were selected and the network was illustrated using the twopi layout.

### Gene expression assay

A TaqMan^®^ RNA-to-Ct Kit and TaqMan^®^ probes (Applied Biosystems, Thermo Fisher Scientific Inc., Waltham, MA) were used for gene expression assays. The probes used are shown in S1 Table, and the *glyceraldehyde-3-phosphate dehydrogenase* gene (*GAPDH*) was used as an internal standard for the assay. Quantitative real-time PCR (qPCR) was performed using an ABI7900HT sequence detection system or a QuantStudio™ 12K Flex Real-Time PCR System (Applied Biosystems). The relative induction of target transcripts was assessed with regard to internal controls according to the manufacturer's instructions. Data were obtained from triplicate measurements, and results are expressed as -fold induction of the expression *vs* controls. Statistical data were calculated using PRISM 5 (GraphPad Software, Inc., La Jolla, CA). When *Ct* was undetermined, data were excluded from the calculation. Data are shown as scatter graphs with means and 95% confidence intervals (CI).

### Protein array for cytokines and chemokines

Multiplex ELISA assay for cytokines and chemokines was performed using a Bio-Plex Pro mouse cytokine multiplex assay kit (Bio-Rad, Hercules, CA) and a Bio-Plex 200 System with high-throughput fluidics as described previously [10]. For statistical analysis, a two-sided Student’s *t*-test was used for comparison of two groups with a Gaussian distribution, while Welch's *t*-test was used for two groups with unequal variances which were estimated by *F* test.

### Immunohistochemistry

Specimens were fixed in 10% formaldehyde in PBS, and were embedded in paraffin. Five μm sections were deparaffinized with a xylene and ethanol series. Sections were incubated with a mouse monoclonal anti-human Myeloid/Histiocyte antigen (S100A8/S100A9) (calprotectin) antibody (clone MAC 387) (Dako Denmark A/S, Glostrup, Denmark) (1:200 dilution), a Vectastain Universal kit (Vector Laboratories Inc.) was used according to the manufacturer’s instructions and staining signals were visualized with diaminobenzidine. Images were recorded using an AX80 microscope equipped with a DP72 CCD camera (Olympus, Tokyo, Japan).

### Antimicrobial assays

Epidermis isolated from 19.5 dpc mice (n = 3) was placed on ice and minced in 0.5 ml 1 M HCl. The specimens were homogenized using a mixer mill MM 300 (Retsch Technology GmbH, Haan, Germany) and were incubated at 4°C for 24 h under rotation. After centrifugation (10,000 x *g*) at 4°C for 10 min, the supernatant was lyophilized and dissolved in 0.3 ml 5 mM MOPS buffer (pH 7.0). The solution was desalted using a PD SpinTrap G-25 (exclusion limit: Mr. 5,000) (GE Healthcare Bio-Sciences, Pittsburgh, PA) and 0.2 μg/μl protein solution was subjected to colony forming unit (CFU) assays as described by Sørensen *et al*. [11]. Bacterial suspensions of 0.2 ml 1.0 x 10^6^ cfu/ml *Staphylococcus aureus* (DSM 346 strain) or *E*. *coli* (K-12 strain) were mixed with 0.1 ml diluted protein solutions or 5 mM MOPS buffer (pH 7.0) as a control and were incubated at 37°C for 3 h. Serially diluted bacterial cultures were plated on Tryptic Soy agar plates, and after incubation at 37°C for 24 h, the number of colonies was counted. For statistical analysis of multiple comparison, one-way ANOVA and Bonferroni post hoc test was used, and a P<0.05 is considered a significant difference.

## Results

### Gene Expression Profiles of *Tgm1*^–/–^Mouse Epidermis

**Fig 1:**
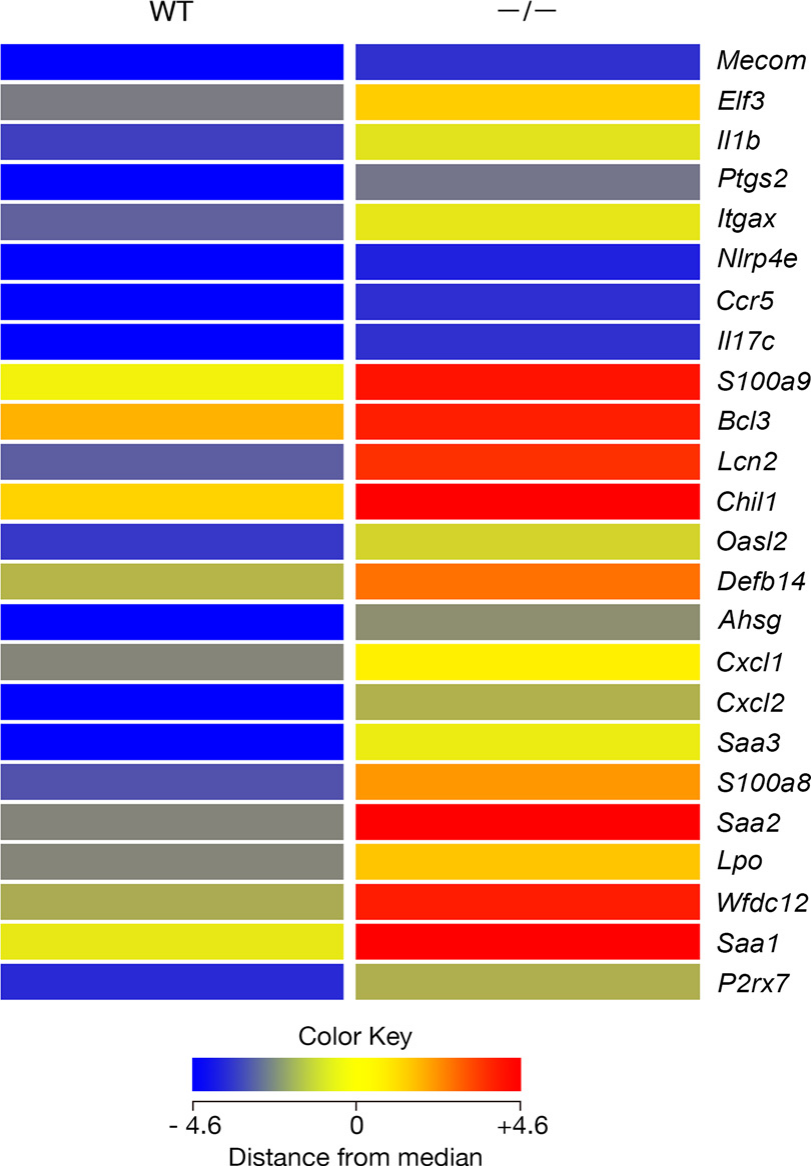
Tiling heat map of the expression of “defense response” genes in wild-type and in *Tgm1*^–/–^epidermis. Each color represents the mean expression of duplicate samples from each type of mouse (19.5 dpc pups, n = 2). Those genes were expressed in *Tgm1*^–/–^mouse epidermis (–/–) more than 5-fold higher than in wild-type epidermis (WT).

The gene expression profile of 19.5 dpc *Tgm1*^–/–^mouse epidermis was compared with that of wild-type mice in duplicate samples, which were analyzed using microarrays. The expression of 2,502 entities was increased more than 2-fold in *Tgm1*^–/–^epidermis *vs* wild-type epidermis, and 1,403 of those entities corresponded to protein-coding genes. Gene ontology (GO) analysis of those entities revealed that the top 15.5% of 90 GO terms with corrected P < 0.05 were related to defense or immune responses. The entities for 177 annotated transcripts showed more than a 5-fold increase in expression in *Tgm1*^–/–^epidermis *vs* wild-type epidermis and those were also subjected to GO analysis. Interestingly, 24 genes from those entities were sorted into the category of defense response (P<2.78E-11; corrected P<1.50E-07) (Fig 1). Of those genes, *S100a8*, *S100a9*, *Defb14*, *Lcn2* and *Wfdc12* encode proteins with antimicrobial activities, S100 calcium binding protein A8 (S100A8) (calgranulin A), S100A9 (calgranulin B), defensin-β 14 (Defb14), the orthologue of human β-defensin 3 (HBD3) (defensin, β 103B), lipocalin 2 (LCN2) (24p3) and WAP four-disulfide core domain 12 (WFDC12), respectively. The expression of other representative skin AMP genes [9], *Ltf* for lactotransferrin (ID_REF: A_52_P15388), *Lyz1* and *Lyz*2 for lysozymes (ID_REF: A_55_P2181738; A_51_P321150), *Serpina1c* for serine (or cysteine) peptidase inhibitor, clade A, member 1C (mouse orthologue of elafin/SKALP) (ID_REF: A_55_P2010301), *Pomc* for α-MSH (ID_REF: A_52_P671543), *Chga* for chromogranin A (mouse orthologue of catestatin) (ID_REF: A_51_P358316), was less than 2-fold in *Tgm1*^–/–^epidermis *vs* wild-type epidermis.

### Gene Expression of AMPs and Their Homologs in *Tgm1*^–/–^Mouse Epidermis

**Fig 2:**
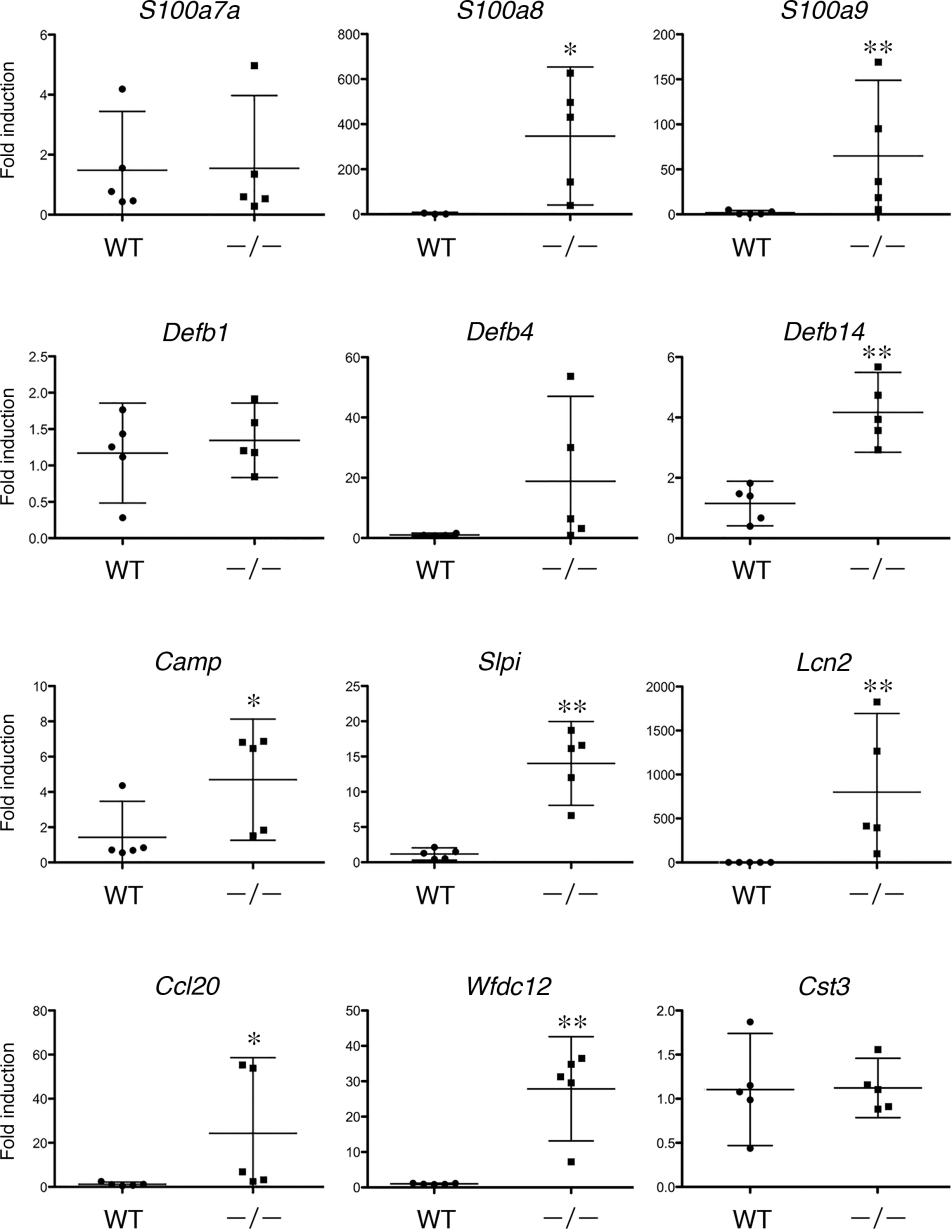
The expression of antimicrobial peptide genes in *Tgm1*^–/–^epidermis *vs* wild-type epidermis. Data were obtained from 5 independent specimens of *Tgm1*^–/–^epidermis (–/–) *vs* wild-type epidermis (WT) (19.5 dpc pups, n = 5), and fold-inductions relative to the expression in wild-type epidermis are plotted with means and bars showing 95% confidence intervals (CI). *, P<0.05; **, P<0.01.

In addition to *S100a8*, *S100a9*, *Defb14*, *Lcn2* and *Wfdc12*, the expression of their homologue(s), *S100a7a*, *Defb1* and *Defb4*, and other AMP genes, *Ccl20* [12], *Slpi*, *Camp* and *Cst3*, was examined by qPCR. As shown in Fig 2, a significantly increased expression of *S100a8*, *S100a9*, *Defb14*, *Camp*, *Slpi*, *Lcn2*, *Ccl20* and *Wfdc12* was found in *Tgm1*^–/–^epidermis *vs* wild-type epidermis. A marked induction of *Defb4* was also observed on average, although it was not statistically significant (P = 0.111) because of individual variability in its expression in *Tgm1*^*–/–*^epidermis. The expression of *A100a7a*, *Defb1* and *Cst3* was not significant between *Tgm1*^*–/–*^and wild-type epidermis.

### Expression of Cytokines and Chemokines in *Tgm1*^–/–^Mouse Skin

**Fig 3:**
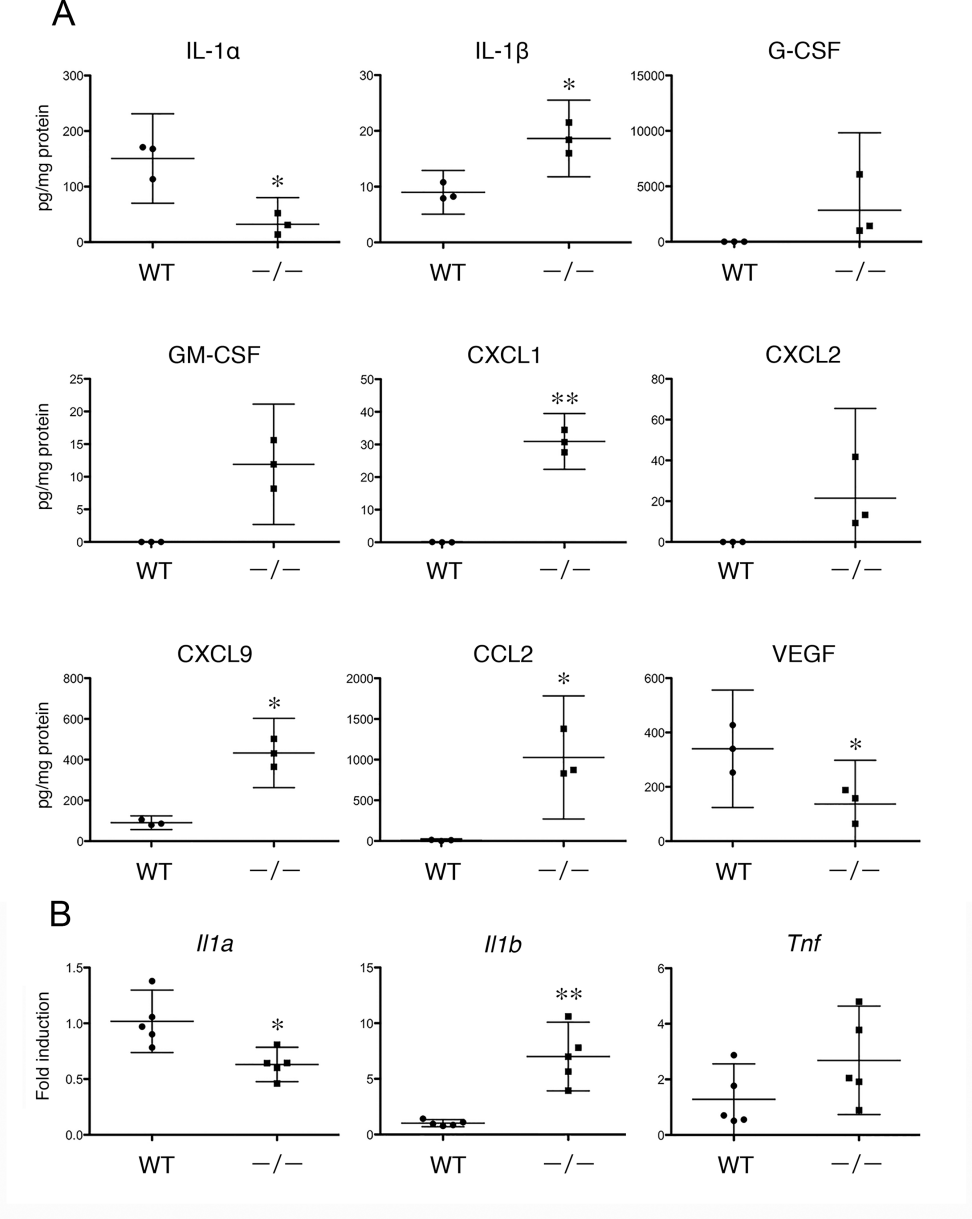
**(A) Protein expression of cytokines and chemokines in wild-type and in *Tgm1***^**–/–**^**skins.** Data were obtained from 3 independent samples of *Tgm1*^–/–^(–/–) and wild-type skin (WT) (19.5 dpc pups, n = 3), and fold-inductions of the mean values of expression in wild-type skins are plotted with means and bars representing 95% CI. *, P<0.05; **, P<0.01. **(B) Gene expression of *Il1a*, *Il1b*, and *Tnf* in wild-type and in *Tgm1***^**–/–**^**epidermis.** Data were obtained from 5 independent specimens of *Tgm1*^–/–^epidermis (–/–) *vs* wild-type epidermis (WT) (19.5 dpc pups, n = 5), and fold-inductions of the mean values of expression in wild-type epidermis were plotted with means and bars representing 95% CI. *, P<0.05; **, P<0.01.

Human β-defensin 2 is stimulated by interleukin-1α (IL-1α) and IL-1β [13], and S100A8-S100A9 protein complex (calprotectin) (L1 protein, MRP-8/MRP-14) is up-regulated by interferon-γ (IFN-γ) and tumor necrosis factor-α (TNF-α) [14] in cultured epidermal cells. In turn, S100A8/A9 induces the expression of cytokines and chemokines such as CXCL1, CXCL2, CXCL3, CXCL8, CCL20, IL-6 and TNF-α in cultured human epidermal keratinocytes (NHEK) [15]. Those *in vitro* findings suggest close interactions of AMPs and those chemokines and cytokines in the skin. To elucidate the induction of cytokines and/or chemokines in *Tgm1*^–/–^skin, 32 cytokines and chemokines were examined using a protein array. As a result, G-CSF (CSF3), GM-CSF (CSF2) and CXCL2 (MIP-2) were not detected in wild-type skin, whereas a marked induction of those proteins was found in *Tgm1*^–/–^skin (Fig 3A). IL-1β, CXCL1 (KC), CXCL9 (MIG) and CCL2 (MCP1) were significantly increased in *Tgm1*^–/–^skin compared with wild-type skin (Fig 3A). In contrast, IL-1α and VEGF were somewhat decreased in *Tgm1*^–/–^skin. IL-2, IL-5, IL-17, CCL4, CCL5, TNF and PDGF were undetected both in wild-type and in *Tgm1*^–/–^skin, and IL-3, IL-4, IL-6, IL-9, IL-10, IL-12, IL-13, IL-15, IL-18, CCL3, CCL11, IFN-γ, b-FGF, LIF and MCSF were not altered between *Tgm1*^–/–^and wild-type skin (S2 Table).

The gene expression of *Il1a*, *Il1b* and *Tnf* in the epidermis was also examined using qPCR (Fig 3B). A significant increase in the expression of *Il1b* was confirmed in *Tgm1*^–/–^epidermis *vs* wild-type epidermis, whereas the expression of *Il1a* was somewhat decreased. The expression of *Tnf* was not significantly different between *Tgm1*^–/–^and wild-type epidermis.

### Expression of EGF Receptor and Its Ligands in *Tgm1*^–/–^Mouse Epidermis

**Fig 4:**
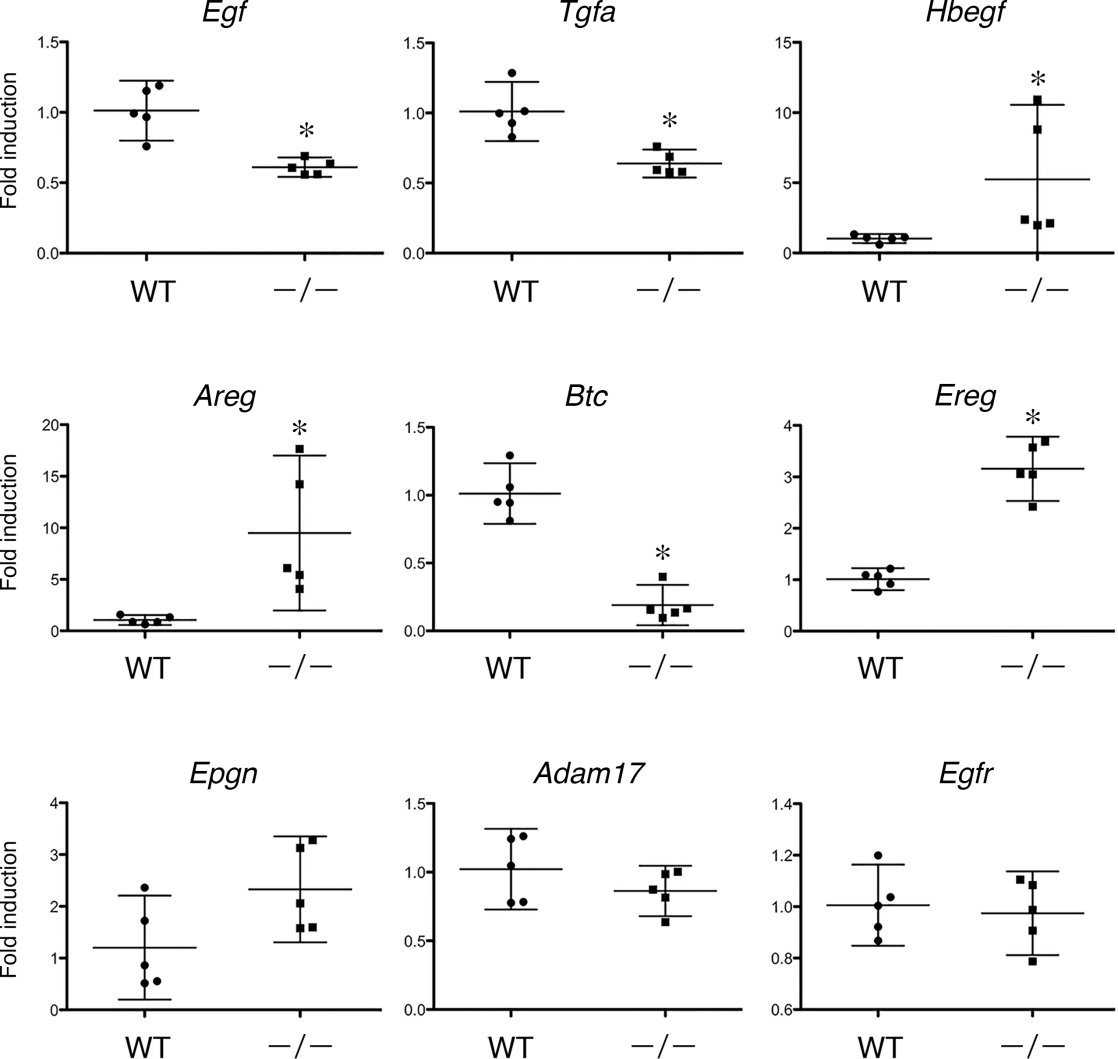
Gene expression of EGFR and its ligands in wild-type and in *Tgm1*^–/–^skin. Data were obtained from 5 independent specimens of *Tgm1*^–/–^epidermis (–/–) *vs* wild-type epidermis (WT) (19.5 dpc pups, n = 5), and fold-inductions of the mean values of expression in wild-type epidermis are plotted with means and bars representing 95% CI. *, P<0.01.

The induction of AMPs such as β-defensin 3, lipocalin 2 and SLPI is thought to be coordinated with transactivation of the EGF receptor (EGFR) in the skin [11]. The cathelicidin antimicrobial peptide activates the EGFR via shedding of a ligand of EGFR, heparin-binding EGF-like growth factor (HB-EGF), in cultured NHEK [16]. Thus, the expression of AMPs may be closely related with EGFR activation in keratinocytes. To elucidate the role of EGFR activation in TGM1 deficiency, the expression of EGFR and its ligands was examined using qPCR in wild-type and in *Tgm1*^–/–^epidermis. As shown in Fig 4, the expression of EGF homolog genes, *Hbegf*, *Areg* and *Ereg* was significantly increased in *Tgm1*^–/–^epidermis *vs* wild-type epidermis. In contrast, the expression of *Egf*, *Tgfa* and *Btc* was somewhat decreased in *Tgm1*^–/–^epidermis. The expression of *Epgn*, *Adam17* and *Egfr* was not altered.

### Antimicrobial activity of *Tgm1*^–/–^epidermis extract

**Fig 5:**
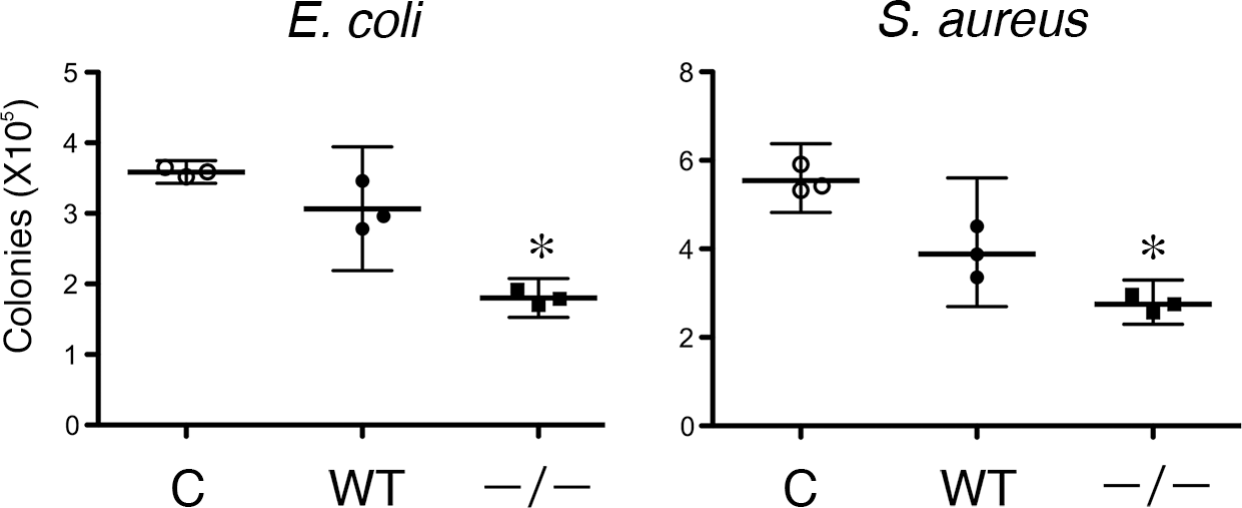
Antimicrobial activities of epidermal extracts. CFU ability was assayed in the presence of 5 mM MOPS buffer (control) (C), an epidermal extract from wild-type mice (WT) or an epidermal extract from *Tgm1*^–/–^mice (–/–) (19.5 dpc pups, n = 3). Dilutions of the extracts were 1/100 and 1/1000 for *E*. *coli* and *S*. *aureus*, respectively. Killing activities against both types of bacteria were more pronounced in the epidermal extract from *Tgm1*^–/–^mice than that from wild-type mice. *, P<0.05 *vs* C and WT.

The up-regulation of molecular signatures for antimicrobial defense responses was highly suggestive of enhanced antimicrobial activity in the *Tgm1*^–/–^epidermis. Therefore, the bacterial killing activity of epidermal extracts was examined using a CFU assay for *E*. *coli* and *S*. *aureus*. As shown in Fig 5, the epidermal extract from *Tgm1*^–/–^mice suppressed CFU for both types of bacteria more than the epidermal extract from wild-type mice. Those results suggest that killing activity against *E*. *coli* and *S*. *aureus* was enhanced in *Tgm1*^–/–^epidermis.

### Expression of S100A8-S100A9 Protein Complex (calprotectin) and Other AMPs and Related Genes in Human Ichthyosis Skin with *TGM1* mutations

**Fig 6:**
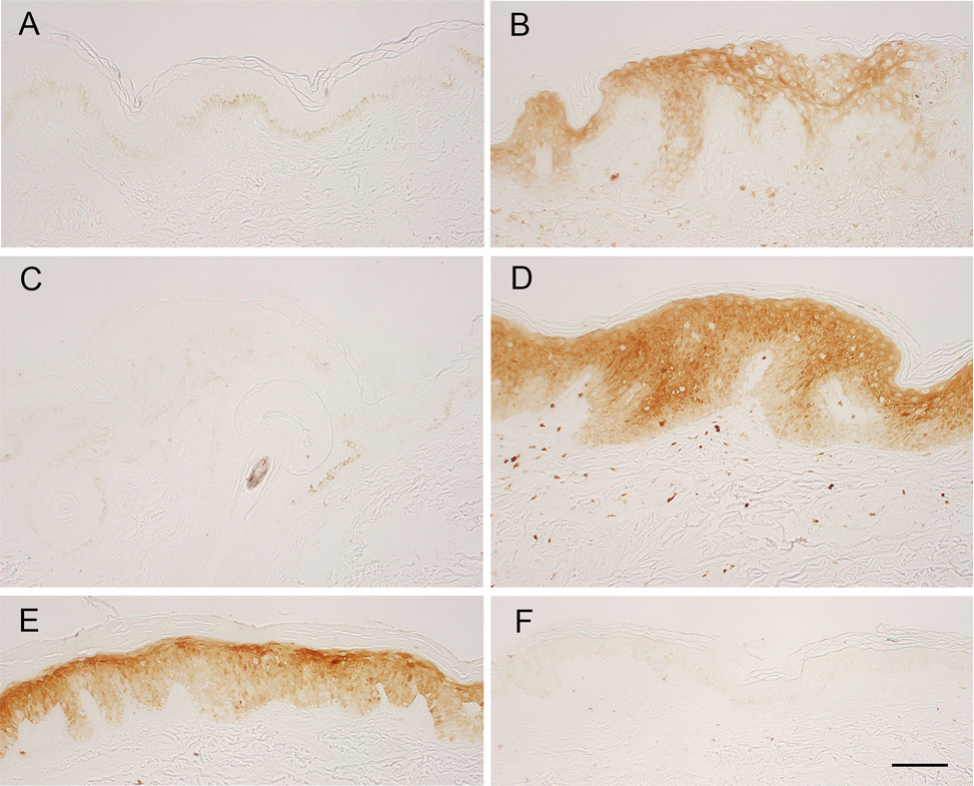
Immunohistochemistry of S100A8-S100A9 protein complex (calprotectin) in human skins. Skin samples from a healthy control (thigh) (A), an anonymous patient with psoriasis vulgaris as a positive control (B), a non-lesional (C) and a lesional skin (D) (lumbar region) from a BSI patient with *TGM1* mutations c.[430G>A];[919C>T], a patient of self-improving collodion ichthyosis with the *TGM1* mutations c.[919C>T]; [1024G>A] (abdomen) (E) and a patient with acquired ichthyosis with no known mutation for congenital ichthyoses (upper arm) (F) were stained with an antibody for calprotectin. The lesional epidermis with the *TGM1* mutations (D, E) was clearly positive for calprotectin. Bar, 100 μm in A-F.

The expression of S100A8-S100A9 protein complex (calprotectin) was examined in the skin of two patients with *TGM1* mutations. One patient had compound heterozygous *TGM1* mutations of c.[430G>A];[919C>T] which leads to p.[G144R];[R307W] in the β-sandwich and core domains of the TGM1 enzyme [17]. That patient shows an unusual phenotype of BSI, in which ichthyosis lesions appear predominantly on warmer parts of the skin. Therefore, we could assess the expression of calprotectin between lesional skin with ichthyosis and non-lesional skin as a control in the same genetic background. Another patient had *TGM1* mutations of c.[919C>T]; [1024G>A], which cause p.[R307W];[R315H] in the core domains of the enzyme. The mutation of p.[R307W] was common with another case of BSI, but the severe generalized ichthyosis was fairly resolved at one year of age, and the phenotype may be compatible with self-improving collodion ichthyosis [18]. For immunohistochemistry of calprotectin, a skin specimen from a patient with psoriasis vulgaris was used as a positive control, because S100A8 and S100A9 are up-regulated in psoriatic epidermis [19]. Furthermore, a skin specimen from a patient with acquired ichthyosis was also used. Coding and splice site mutations in known genes responsible for ichthyoses were excluded by target re-sequencing of genomic DNA from the patient. As shown in Fig 6, staining for calprotectin was negative in the epidermis of healthy skin (Fig 6A), in non-lesional skin of the patient with BSI (Fig 6C), and in skin from a patient with acquired ichthyosis (Fig 6F). In contrast, calprotectin was intensely positive in the epidermis of lesional ichthyosis skins with those *TGM1* mutations (Fig 6D and 6E), as well as in lesional epidermis of psoriasis (Fig 6B). Some dermal infiltrates were also positive in those sections.

**Fig 7:**
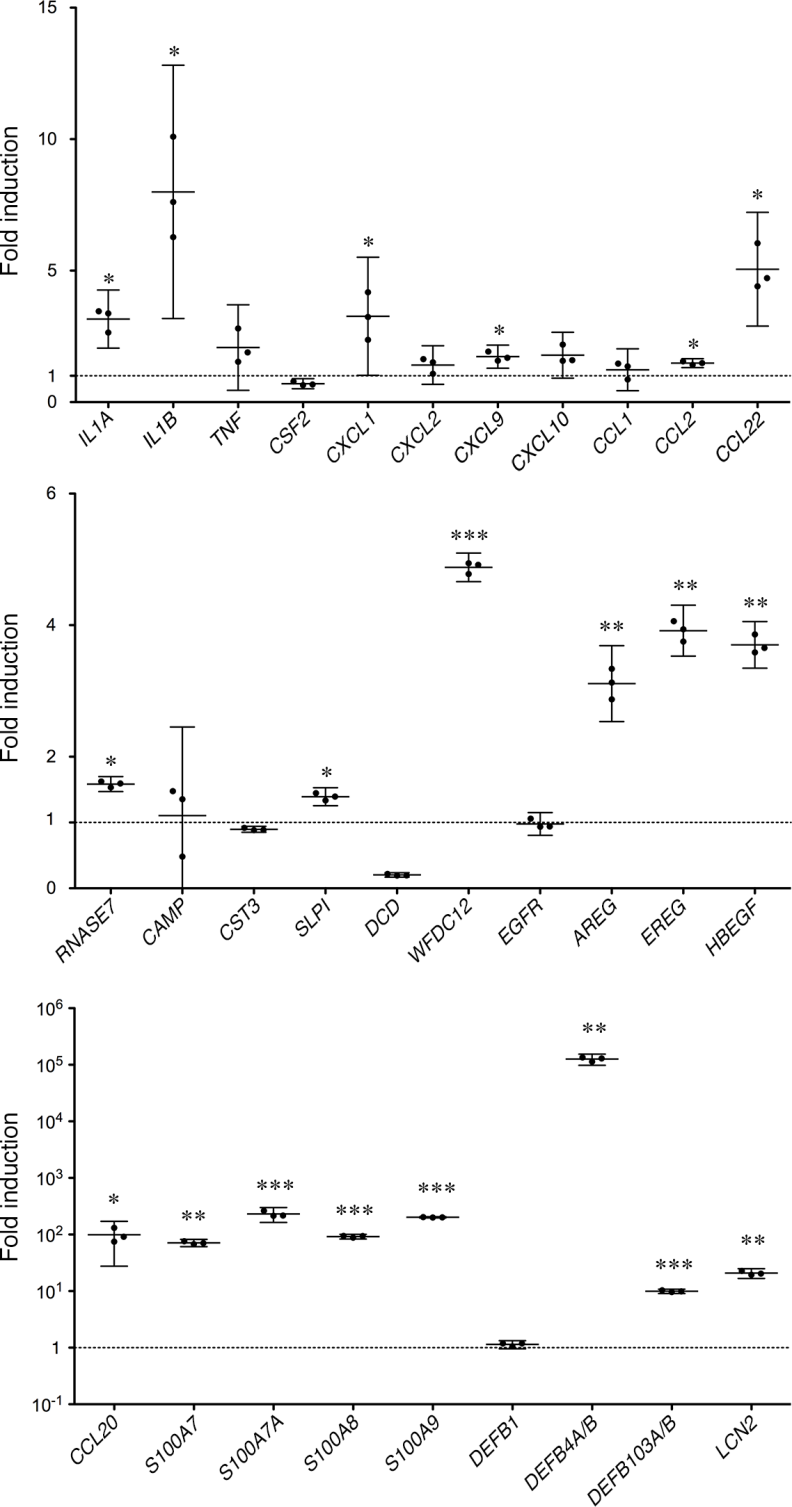
Gene expression of antimicrobial peptides, cytokines, chemokines and EGFR and its ligands in the lesional skin of a BSI patient with *TGM1* mutations c.[430G>A];[919C>T]. Fold-inductions of each designated gene in the lesional skin *vs* non-lesional skin were plotted with means and bars representing 95% CI. The gene expression levels of IL-1α, IL-1β, CXCL1, CXCL9, CCL2, CCL22, RNASE7, SLPI, WFDC12, AREG, EREG and HBEGF were significantly increased from about 1.4 to 8-fold on average, and levels of CCL20, S100A7, S100A7A, S100A8, S100A9, DEFB4A/B, DEFB103A/B and LCN2 were markedly increased and ranged from 10-fold to 10^5^-fold in the lesional skin *vs* non-lesional skin. *, p<0.05; **, p<0.005; ***, p<0.0005.

Based on analysis using *Tgm1*^–/–^mouse skin, the gene expression of AMPs, cytokines, chemokines and EGFR ligands was examined in the lesional and non-lesional skin from the patient with BSI using qPCR (Fig 7). The expression of *IL1Α*, *IL1Β*, *CXCL1*, *CXCL9*, *CCL2*, *CCL22*, *RNASE7*, *SLPI*, *WFDC12*, *AREG*, *EREG* and *HBEGF* was increased from about 1.4- to 8-fold on average in the lesional ichthyosis skin. Furthermore, the gene expression of *CCL20*, *S100A7*, *S100A7A*, *S100A8*, *S100A9*, *DEFB4A/B*, *DEFB103A/B* and *LCN2* was markedly increased and ranged from 10-fold to 10^5^-fold in the lesional skin. In contrast, the gene expression of *CSF2*, *CST3* and *DCD* was decreased. The expression of *TNF*, *CXCL2*, *CXCL10*, *CCL1*, *CAMP*, *EGFR* and *DEFB1* was not significantly altered.

## Discussion

In the present study, we demonstrate that AMP genes encoding S100A8, S100A9, defensin β-3 (Defb14, DEF103A/B), SLPI, WFDC12, LCN2 and CCL20 are highly up-regulated in *Tgm1*^–/–^epidermis. Importantly, in accordance with the induction of those genes, the bacteriocidal activities of *Tgm1*^–/–^epidermis against *E*. *coli* and *S*. *aureus* are actually enhanced. Furthermore, those genes are also up-regulated in the skin of human TGM1 deficiencies.

Because of the neonatal lethality of *Tgm1*^–/–^mice, the epidermis at 19.5 dpc, just before birth, was used to analyze the gene expression profile. The up-regulated expression of those genes suggests that the process of their induction in *Tgm1*^–/–^mouse skin occurs before birth in utero. The stratum corneum barrier is generated around 16 dpc and the barrier formation is totally defective in *Tgm1*^–/–^mice [6]. The defective development of the stratum corneum might trigger the induced expression of those AMP genes. Alternatively, the exposure of the immature epidermis to amniotic fluid in utero might permit the gene expression of AMPs. Huebner *et al*. [20] have reported that the Nrf2/Keap1 pathway is activated by amniotic fluid to compensate for the epidermal barrier defect in loricrin knockout mice. In our microarray data, the expression of *Keap1* (ID_REF:A_51_P438666) was decreased about 27% on average in *Tgm1*^–/–^epidermis, and Nrf2-responsive genes, such as *Slpi*, *Krt6a* (ID_REF:A_52_P104658), *Sprr2d* (ID_REF: A_51_P435588) and *Rptn* (ID_REF:A_52_P523146), were markedly induced, while *Sprr2h* (ID_REF:A_66_P138462) and *Srxn1* (ID_REF:A_55_P2033120) were induced about 2-fold. On the other hand, other Nrf2-responsive genes, *Prdx1* (ID_REF:A_55_P2147427; A_66_P119421) and *Gclc* (ID_REF:A_51_P365019) were only slightly induced and the expression of *Nqo1* (ID_REF:A_51_P424338) and *Gstp2* (*Gst3*) (ID_REF:A_55_P1957038;A_55_P2008704) was decreased in *Tgm1*^–/–^epidermis. Thus, the effect of the Nrf2/Keap1 pathway on those genes, if any, might be selective in *Tgm1*^–/–^mice.

S100A8 and S100A9 are members of the S100 family of proteins, and calprotectin, a heterodimer of those proteins, has an innate, antimicrobial activity in epithelia [21]. The pronounced gene expression of S100A8 and S100A9 is a feature of *Tgm1*^–/–^epidermis and in the lesional skin of BSI with *TGM1* mutations c.[430G>A];[919C>T]. The induction of calprotectin is seen in all nucleated layers of the lesional epidermis of ARCIs with different TGM1 mutations. S100A8 and S100A9 are induced abundantly in hyperproliferative states of the epidermis [22], and in cultured NHEK, those proteins are induced by IL-1α, IL-6, IL-8, TNF-α, IFN-α and/or IFN-γ [14, 15].

IL-1α is increased in ARCI and has been postulated to be a key cytokine involved in the hyperkeratosis in TGM1 deficiency using a rat organotypic culture model and rat Tgm1 siRNA [23]. In accordance with that study, the gene expression of IL-1α was induced in the lesional skin of BSI with the TGM1 mutations in the present study. However, the gene and/or protein expression of IL-1β was also noted in the skin of *Tgm1*^–/–^mice and in the lesional BSI skin. The up-regulation of genes for other cytokines, including IL-6, IL-8, TNF-α, IFN-α and IFN-γ, was not evident in *Tgm1*^–/–^mice. Therefore, IL-1β also plays a role as an inducer of S100A8 and S100A9 in TGM1 deficiency.

In those mouse and human TGM1 deficiencies, the gene expression of β-defensin 3 (Defb14, DEF103A/B) was found. β-Defensin 3 has a broad spectrum antimicrobial activity, in particular, against *S*. *aureus* [24, 25]. β-Defensin 3 is increased in psoriatic skin, but is reduced in atopic dermatitis skin [26]. In cultured human primary keratinocytes, transcripts of β-defensin 3 are induced by TNF-α, IFN-γ and IL-1β [27]. Hence, the induction of IL-1α/β might be involved in the expression of β-defensin 3 in the skin of TGM1 deficiency. Insulin-like growth factor I and TGF-α can also induce β-defensin 3 and other AMPs in cultured human keratinocytes [28]. Recently, Gschwandtner *et al*. [29] reported that the expression of AMPs, including β-defensin 3, is high in fetal skin and they postulated that the expression is controlled by a histone demethylase, JMJD3, now named KDM1 lysine (K)-specific demethylase 6B (KDM6B). However, *Kdm6b* (ID_REF: A_55_P2030080), *Igf1* (ID_REF: A_55_P2031631; A_55_P2031636; A_55_P2085974; A_55_P2085979; A_55_P2085984) as well as *Tgfa* was not induced in the epidermis of *Tgm1*^–/–^mice in our microarray data, and therefore it is unlikely that processes involving JMJD3 and those growth factors induce the expression of AMPs in *Tgm1*^–/–^mice.

SLPI (Secretory leukocyte protease inhibitor) is a small cationic protein with a serine protease inhibitor activity. SLPI inhibits a variety of proteases, such as trypsin, chymotrypsin, leukocyte elastase and cathepsin G. However, the antimicrobial activity of SLPI may be dependent on its cationic nature, but not necessarily on its anti-protease activity [30]. SLPI is up-regulated in the epidermis of psoriasis patients and in injured skin and is induced during the proliferation of keratinocytes [31]. On the other hand, WFDC12 is a member of the whey acidic protein (WAP) family [32] and WFDC12 levels in bronchoalveolar lavage fluid are increased in inflammatory respiratory conditions [33]. The roles of SLPI and Wfdc12 in the skin are not fully understood, but the up-regulation of those proteins in TGM1 deficiency might contribute to innate defense responses of the skin through anti-protease, anti-microbial and/or anti-inflammatory activities.

LCN2 is a neutrophil gelatinase-associated lipocalin (NGAL), which was discovered as a protein associated covalently with neutrophil gelatinase [34]. LCN2 has a potent bacteriostatic activity due to its interference with bacterial ferric siderophore-mediated iron acquisition [35]. LCN2 is induced in the epidermis by skin injury [28] and is increased in lesional skin of patients with psoriasis, pityriasis rubra pilaris and chronic eczema, but not in those with acute eczema or atopic dermatitis [36, 37]. In human HaCaT keratinocytes, IL-1α induces LCN2 as well as S100A7, S100A8, S100A9 and SLPI [13]. LCN2 is regulated by the transcription factor Tcf3 during wound healing of the skin [38]. However, the expression of Tcf3 was not induced in *Tgm1*^–/–^epidermis in our microarray analysis (ID_REF: A_51_P394471; A_55_P1975354). As suggested recently in a psoriasis model [39], LCN2 may play a role in enhancing other AMPs in the skin in concert with other cytokines/chemokines.

CCL20 (macrophage inflammatory protein-3α; MIP-3α) is a CC chemokine released from keratinocytes and other types of cells in the skin. CCL20 is chemotactic for CLA^+^ memory T cells and dendritic cells expressing CC chemokine receptor-6 [40]. CCL20 also shows a strong antibacterial activity against *E*. *coli* and *S*. *aureus* [12]. CCL20 is up-regulated in psoriasis and in activated keratinocytes of cutaneous injury and of UVB irradiated skin [40, 41]. The expression of CCL20 in keratinocytes is induced by TNF-α, IL-1β, CD40 ligand, IFN-γ and IL-17 [40], and therefore IL-1β might be an inducer of CCL20 in TGM1 deficiency.

Besides the physical stresses of skin injury and UVB irradiation and the stimulation by cytokines, AMPs are also regulated downstream of the EGFR signaling pathway [42]. Some AMPs, including DEFB4, CCL20 and S100A7, are synergistically induced by signals from the EGFR and IL-1 in keratinocytes [43]. In *Tgm1*^–/–^skin, the up-regulation of EGFR ligand genes, *Hbegf*, *Areg* and *Ereg*, in the epidermis is suggestive of a condition in which AMPs are more easily up-regulated. Interestingly, this condition is also maintained in the lesional skin of a BSI patient with the TGM1 mutation and possibly contributes to hyperplasia of the epidermis in the ichthyosis. This setting is similar to skin injury in which AMPs are induced with the activation of EGFR via HB-EGF in human skin [11], although direct evidence for EGFR activation was not assessed in the preset study.

**Fig 8:**
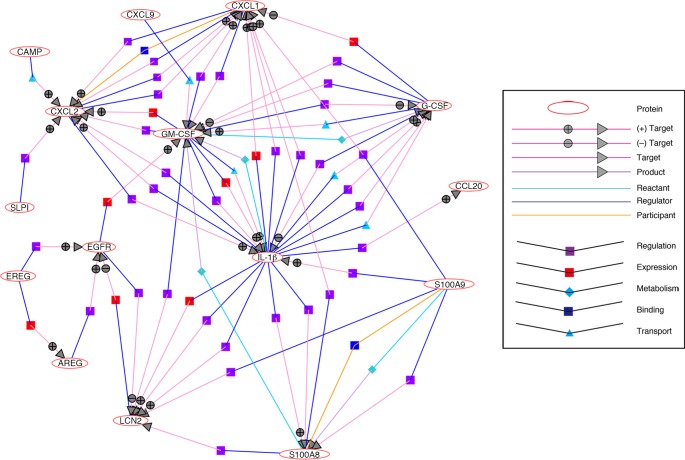
Network and interactions of molecular signatures up-regulated in *Tgm1*^–/–^skin. Genes for alarmins or antimicrobial peptides S100A9, S100A8, LCN2, SLPI, CAMP and CCL20 are induced along with IL-1β and other cytokines GM-CSF (CSF2) and G-CSF (CSF3) and chemokines CXCL1, CXCL2, and CXCL9 in *Tgm1*^–/–^skin, where EGFR may be activated with the induction of its ligand genes for EREG and AREG.

In TGM1 deficiency, the CE of the stratum corneum is lost and skin barrier function is disrupted with irregular arrangements of intercellular lipids [3, 6, 7]. Marionnet *et al*. found that S100A8 and S100A9 are induced in healthy human skin by light stresses such as tape stripping [44]. The induction of those AMP genes is not so intense as in the *Tgm1*^–/–^epidermis and in the lesional skin of BSI with the TGM1 mutations. However, the response of those proteins to stress seems highly sensitive. A cutaneous injury drives further innate immune responses in accordance with the activation of other AMPs, cytokines, chemokines, and EGFR. Like injury stresses to the epidermis, the severe morphological, biochemical and functional derangements in the *Tgm1*^–/–^epidermis and in ARCI might be sufficient to induce those AMPs as “alarmins”. A possible network and interactions of those AMPs, cytokines/chemokines and EGFR and it ligands are illustrated in Fig 8, based on analysis using NLP.

Roth *et al*. [45] reported a prenatal increase of S100A8, S100A9 and IL-18 in keratin 1 knockout mouse (*Krt1*^–/–^) skin and they proposed a keratinocyte-autonomous inflammatory process that is partially dependent on IL-18. The activation of IL-18 in the skin induces super Th1 cells which produce both Th1- and Th2-type inflammation in mice [46]. In contrast to *Krt1*^–/–^skin, *Tgm1*^–/–^skin revealed no increase in IL-18 protein (S2 Table), although *Krt1*^–/–^mice were examined in a mixed genetic background 129/Ola6C57BL/6 [45] and the difference in IL-18 induction between *Krt1*^–/–^and *Tgm1*^–/–^skins might depend on the genetic background of those mice.

Recently, an entombment of cathelicidin and human β-defensin 2 within the cytoplasm of corneocytes was suggested to be a risk for secondary infection in Harlequin ichthyosis and epidermolytic ichthyosis because of the exocytosis impairment of lamellar granules [47]. A few persistent cutaneous fungal infections in LI have been documented, but those are rare cases, and little is known about the frequency of infection in ARCI patients with TGM1 mutations. The secretion of lamellar granule contents is disrupted in *Tgm1*^–/–^skin [6] and therefore it might be possible that some of the abundant AMPs are functionally abortive, although this could not be precisely assessed in the present study.

The mechanisms for the induction of the molecular signatures for antimicrobial and innate defense responses in TGM1 deficient skin are possibly very complex processes. As a speculation, the TGM1 deficiency causes developmental immaturity in the stratum corneum, and, as occurs in injured skin, the structural defects may autonomously and constitutively induce a set of “alarmins” such as S100A8 and S100A9 and other AMPs with the release of EGFR agonists and several cytokines/chemokines in the epidermis, along with the stimulation by exposure to amniotic fluid in utero and xenobiotic stresses after birth. Those processes may serve as a functional compensation for the defective skin barrier in TGM1 deficiency.

## Conclusion

The present study reveals that the molecular signatures for antimicrobial and innate defense responses are up-regulated in skin with a TGM1 deficiency, including human ARCIs. The activation of those molecular signatures might be a characteristic process underlying the phenotype of ARCI to reinforce the defective skin barrier.

## Supporting Information

S1 Table Probe list for gene expression assay. (DOCX)

S2 Table Expression of other cytokines/chemokines in mouse skin. (DOCX)
