# Supplementary material for: Activation of Molecular Signatures for Antimicrobial and Innate Defense Responses in Skin with Transglutaminase 1 Deficiency
Source: PLoS One. 2016 Jul 21;11(7):e0159673. doi: 10.1371/journal.pone.0159673 (PMC4956052; doi:10.1371/journal.pone.0159673)
Supplement: S1 Table — (DOCX) [file pone.0159673.s001.docx]

**S1 Table. Probe list for gene expression assay.**

| Mouse | | Human | |
| --- | --- | --- | --- |
| Gene | Probe | Gene | Probe |
| *Adam17* | Mm00456428_m1 | *AREG* | Hs00950669_m1 |
| *Areg* | Mm00437583_m1 | *CAMP* | Hs00189038_m1 |
| *Btc* | Mm00432137_m1 | *CCL1* | Hs00171072_m1 |
| *Camp* | Mm00438285_m1 | *CCL2* | Hs00234140_m1 |
| *Ccl20* | Mm01268754_m1 | *CCL20* | Hs01011368_m1 |
| *Cst3* | Mm00438347_m1 | *CCL22* | Hs01574247_m1 |
| *Defb1* | Mm00432803_m1 | *CSF2* | Hs00929873_m1 |
| *Defb14* | Mm00806979_m1 | *CST3* | Hs00264679_m1 |
| *Defb4* | Mm00731768_m1 | *CXCL1* | Hs00605382_gH |
| *Egf* | Mm00438696_m1 | *CXCL10* | Hs01124251_g1 |
| *Egfr* | Mm00433023_m1 | *CXCL2* | Hs00601975_m1 |
| *Epgn* | Mm00504344_m1 | *CXCL9* | Hs00171065_m1 |
| *Ereg* | Mm00514794_m1 | *DCD* | Hs00364976_m1 |
| *Gapdh* | Mm99999915_g1 | *DEFB1* | Hs00608345_m1 |
| *Hbegf* | Mm00439306_m1 | *DEFB103A/B* | Hs04194486_g1 |
| *Il1a* | Mm00439620_m1 | *DEFB4A/4B* | Hs00175474_m1 |
| *Il1b* | Mm00434228_m1 | *EGFR* | Hs01076090_m1 |
| *Lcn2* | Mm01324470_m1 | *EREG* | Hs00914313_m1 |
| *Ptgs2* | Mm00478374_m1 | *GAPDH* | Hs99999905_m1 |
| *S100a7a* | Mm01218201_m1 | *HBEGF* | Hs00181813_m1 |
| *S100a8* | Mm00496696_g1 | *IL1A* | Hs00174092_m1 |
| *S100a9* | Mm00656925_m1 | *IL1B* | Hs01555413_m1 |
| *Slpi* | Mm00441530_g1 | *LCN2* | Hs01008571_m1 |
| *Tgfa* | Mm00446232_m1 | *RNASE7* | Hs00922963_s1 |
| *Tnf* | Mm00443258_m1 | *S100A7* | Hs01923188_u1 |
| *Wfdc12* | Mm00454744_g1 | *S100A7* | Hs00374264_g1 |
|  |  | *S100A7A* | Hs00752780_s1 |
|  |  | *S100A8* | Hs00610058_m1 |
|  |  | *S100A9* | Hs00610058_m1 |
|  |  | *SLPI* | Hs00268204_m1 |
|  |  | *TNF* | Hs00174128_m1 |
|  |  | *WFDC12* | Hs00374115_g1 |
