# Supplementary material for: Activation of Molecular Signatures for Antimicrobial and Innate Defense Responses in Skin with Transglutaminase 1 Deficiency
Source: PLoS One. 2016 Jul 21;11(7):e0159673. doi: 10.1371/journal.pone.0159673 (PMC4956052; doi:10.1371/journal.pone.0159673)
Supplement: S2 Table — (DOCX) [file pone.0159673.s002.docx]

**S2 Table. Expression of other cytokines/chemokines in mouse skin.**

| Protein | Mean (n=3) | | 95% CI | P value |
| --- | --- | --- | --- | --- |
|  | Wild-type | *Tgm1*^-/-^ |  |  |
| IL-3 | 0.067 | 0.077 | -0.076 to 0.056 | 0.580 |
| IL-4 | 0.820 | 0.880 | -0.861 to 0.7408 | 0.649 |
| IL-6 | 0.030 | 2.883 | -8.842 to 3.135 | 0.177 |
| IL-9 | 131.1 | 105.9 | -108.1 to 158.6 | 0.501 |
| IL-10 | 3.783 | 2.437 | -4.696 to 7.389 | 0.439 |
| IL-12p40 | 3.783 | 2.437 | -4.696 to 7.389 | 0.439 |
| IL-12p70 | 20.91 | 12.28 | - 6.920 to 23.56 | 0.131 |
| IL-13 | 29.10 | 41.23 | -37.14 to 12.87 | 0.172 |
| IL-15 | 1.947 | 10.63 | -27.39 to 10.02 | 0.184 |
| IL-18 | 8.530 | 5.770 | -4.966 to 10.49 | 0.264 |
| CCL3 | 3.867 | 57.81 | -114.6 to 6.756 | 0.062 |
| CCL11 | 6.720 | 80.80 | -193.2 to 45.00 | 0.116 |
| IFN-γ | 0.783 | 2.540 | -3.896 to 0.382 | 0.072 |
| b-FGF | 152.3 | 175.5 | -198.4 to 152.1 | 0.627 |
| LIF | 1.237 | 19.78 | -73.80 to 36.72 | 0.286 |
| MCSF | 99.07 | 164.2 | -159.3 to 28.97 | 0.097 |
